# Ethical Conduct of Research with Migrants and Refugees: A Systematic Qualitative Review of Ethics Guidelines

**DOI:** 10.1007/s10903-026-01894-z

**Published:** 2026-04-14

**Authors:** Rajshree Thapa, Davoud Pourmarzi, Sarah Cherian, Christine Phillips, Margaret Kay, Ian Pieper, Raylynn Benn, Chiedza Malunga, Thomas Volkman, Holly Seale, Budiadi Sudarto, Bryan Mukandi, Jacqueline Boyle

**Affiliations:** 1 Eastern Health Clinical School, Monash University, 5 Arnold Street, Box Hill, 3128 Melbourne, Australia; 2 National Centre for Epidemiology and Population Health, Australian National University, Canberra, Australia; 3 Department of Refugee and Global Health and General Paediatrics, Perth Childrens Hospital, Perth, Australia; 4 Division of Paediatrics, University of Western Australia, Perth, Australia; 5 School of Medicine and Psychology, Australian National University, Canberra, Australia; 6 Companion House Medical Service, Australian Capital Territory, Australia; 7 General Practice Clinical Unit, Faculty of Medicine, The University of Queensland, Queensland, Australia; 8 School of Public Health, University of Sydney, New South Wales, Australia; 9 Migrant and Refugee Health Partnership, New South Wales, Australia; 10 School of Population Health, UNSW Sydney, Sydney, Australia; 11 Ananda Training and Consultancy, Melbourne, Australia; 12 School of Humanities and Social Inquiry, University of Wollongong, New South Wales, Australia

**Keywords:** Ethics, Guidelines, Research, Refugee, Migrants

## Abstract

There is increasing consensus across communities, health and social care practitioners and researchers in Australia of the need for specific ethical guidelines for research with migrants and refugees. We aim to identify existing documents providing ethics guidance for research with people from migrant and refugee communities, to appraise their quality, synthesise recommendations and identify gaps where additional guidance is required. This systematic review adheres to the Preferred Reporting Items for Systematic Reviews and Meta-Analyses (PRISMA), 2020 guidelines. Appraisal of Guidelines, Research and Evaluation (AGREE) II was used to assess the quality of guidelines. Data sources include OVID Medline, EMBASE, CINAHL and Google Scholar from the database until June 2025. We identified 18 guidelines that fulfilled the eligibility criteria. The six principles identified to meet the specific needs of research with people from migrant and refugee communities were: (i) non-maleficence, the principle of “do no harm”; (ii) empowerment and community participation; (iii) social, health and scientific value of the research; (iv) respect for rights and dignity; (v) cultural sensitivity; and (vi) informed consent. The guidance documents were primarily focused on non-maleficence (avoiding the harms of research) rather than inclusive and collaborative research practices. There was insufficient information available in each guideline to evaluate quality across the different domains of the AGREE II. There are limited comprehensive, good-quality ethics guidelines for research with migrant and refugee communities. There is a clear opportunity to develop with communities, authoritative guidance that incorporates relevant recommendations from existing guidelines, addressing gaps noted, and includes implementation plans.

*Registration: *PROSPERO, CRD42024473285.

**Supplementary Information:**

The online version contains supplementary material available at 10.1007/s10903-026-01894-z.

## Background

Globally, the heightened complexity of geopolitical affairs and economic forces, climate-induced challenges and systematic violations of human rights have driven unprecedented numbers of people to migrate to new environments, often crossing country borders [1]. In Australia and elsewhere, migrants and refugees are frequently under-enumerated, not included in routine data collection, and not incorporated into descriptive and translational research and clinical trials [2, 3]. Research with migrant and refugee communities provides the evidence base to identify and address health and social inequalities and improve access to health services. The Rabat Declaration in June 2023 [4] called for investment in high-quality research to meet the health needs of people on the move. The pioneering statement of research priorities for migrants and refugees, released by the World Health Organisation (WHO) in November 2023, highlighted as one of their five priorities, the need for new ways to collaborate in research and to translate this into policy and practice [5]. Consequently, the interest and volume of research concerning people from migrant and refugee backgrounds has increased in Australia and internationally [6]. It is important to recognise in research that migrant and refugee populations are heterogeneous, encompassing varied, often intersecting, backgrounds across age, socioeconomic status, culture, religion, ethnicity, language, gender and sexuality [7, 8]. Competency in the language(s) of the receiving country and visa status may impact upon participation in research, community connections and visibility and directly and indirectly impact health and healthcare [6–9]. Lack of meaningful engagement and unclear benefits to the communities can hinder participation in research. For individuals with uncertain visa conditions, the fear of deportation, detention or cancelling of visas add further dimensions to their reluctance to participate in research [10].

Ethical research that involves migrant and refugees may seek to (i) understand lived experiences of health and well-being; (ii) identify baseline health needs (which may be culturally and ethnically nuanced); (iii) determine gaps in service delivery and public health interventions (iv) develop evidence-based policies and interventions for improved equity, health outcomes and social care [6], and (v) explore the political and commercial determinants of health. In this publication, we review current guidelines to protect autonomy and wellbeing, whilst simultaneously ensuring equity and inclusivity across all levels of the research continuum for migrants and refugees. A recent systematic review by Davidson et al. in 2024 [11] addressed national and international guidelines for ethical considerations in research with refugees and asylum seekers. While the review focused on understanding vulnerability, we synthesise key principles and recommendations to highlight a strengths-based approach with community involvement in research and extend the scope to include migrant populations. Our systematic review sought to identify existing guidelines, appraise quality, synthesise the recommendations, and identify gaps in current guidelines pertaining to knowledge, practice and research implementation.

## Methods

### Stakeholder Engagement

A Technical Advisory Group (TAG) was formed to guide the protocol, discuss the results and inform our conclusions. The group included young people, members of Lesbian, Gay, Bisexual, Transgender, Queer/Questioning, Intersex, and Asexual + (LGBTQIA+) communities, clinicians, academics involved in research with migrant and refugee communities, community organisations and those with lived experience [12].

## Search Strategy and Inclusion Criteria

This systematic review adheres to the Preferred Reporting Items for Systematic Reviews and Meta-Analyses (PRISMA) 2020 guidelines [13]. The review included peer-reviewed articles and grey literature (e.g., websites and reports) in the English language. Four databases: OVID Medline, EMBASE, CINAHL and Google Scholar were searched from inception until June 2025 using the key terms (Ethics OR Consent) AND (Protocol OR Guideline OR Consideration OR Recommendations) AND (Migrant OR Refugee OR Refugee background OR Displaced population). The search terms were developed in consultation with a librarian from Monash University and technical working group members. A detailed search strategy is outlined in Supplementary Table S1**.** Reference lists of included literature were manually screened. We searched the websites of relevant organisations such as the United Nations High Commission for Refugees (UNHCR), International Organisation for Migration (IOM), WHO and the Pan American Health Organisation, Refugee Council of Australia, and Migrant and Refugee Health Partnership. The search included all publications available in, or translated into, the English language. We included all the guidelines for research with refugees, or migrants or displaced population that were published in English. Guidelines were not restricted by country income, and we included all guidelines from high, middle- or low-income countries. We excluded individual research papers on issues and challenges and researchers’ experiences. Thesis and book chapters were also excluded. Similarly, documents related solely on health service delivery than research were also excluded.

## Selection of Articles

**Fig. 1:**
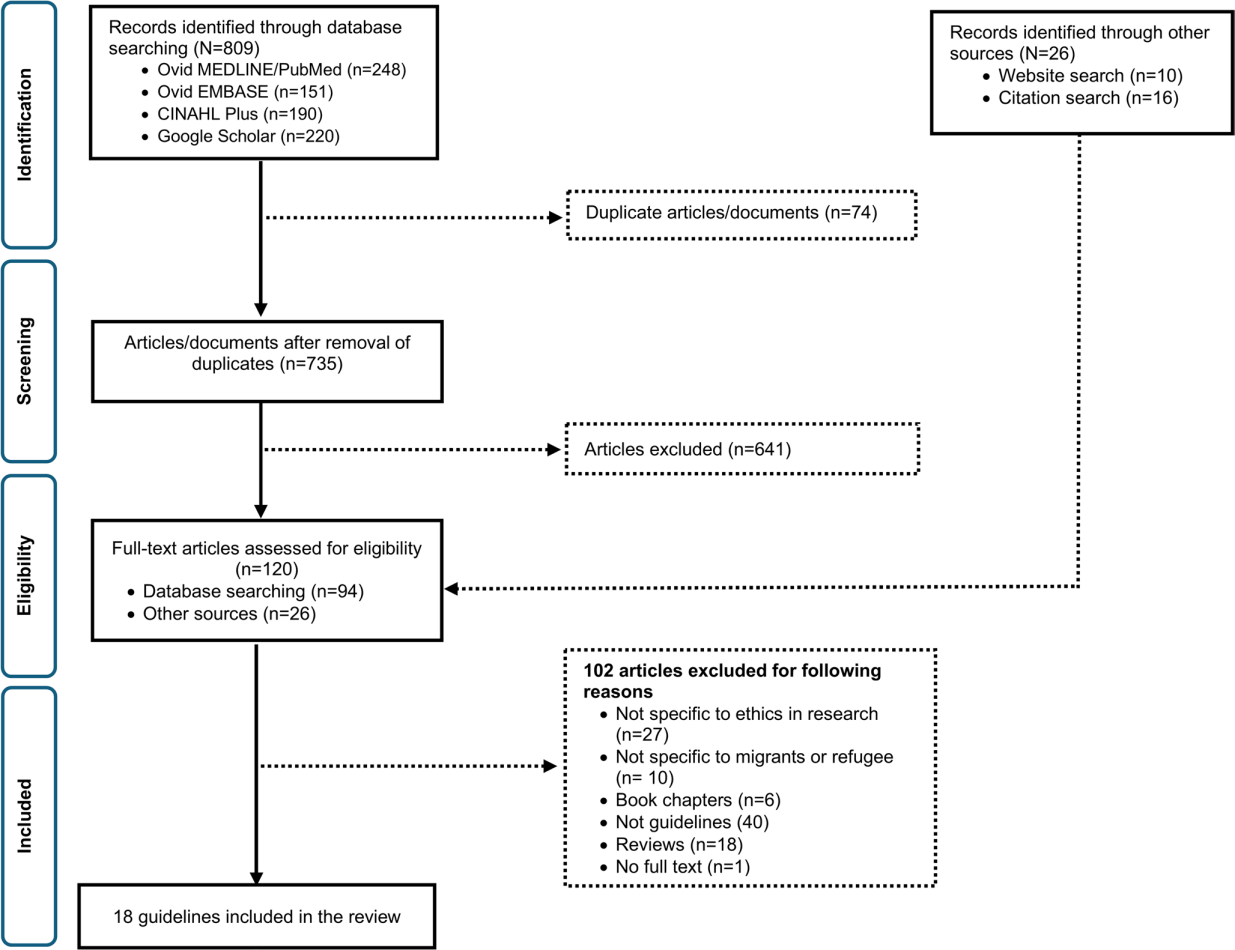
Screening and selection of guidelines

RT searched the electronic databases and subsequently imported the retrieved articles into an online reference manager (*Covidence*), removing duplicates. RT, DP and JB screened the titles and abstracts to assess the eligibility of the articles against the inclusion criteria and undertook full-text review to determine suitability for inclusion. Full-text articles/documents included those retrieved from the title and abstract screening and those identified from other sources including from grey literature search **(**Fig. 1**)**.

## Data Extraction and Management

A PRISMA flow chart (Fig. 1) was used to visualise and summarise the screening process for articles and the grey literature. Data extracted from each document included the title of the guideline, authors and/or organisation, target population (including age), inclusion of refugees or migrants in the guideline, models or frameworks, implementation plan and summary of principles or recommendations. The Appraisal of Guidelines for Research and Evaluation *(AGREE) II* [14], an international tool to assess the quality and reporting of practice guidelines was used to evaluate the quality of each guideline. This tool consists of a checklist with 23 different items across the six main domains. Three reviewers (RT, DP, JB) individually appraised each guideline. Scoring and assessment of quality was not possible due to a lack of information in the documents. Instead, a descriptive summary of quality, framed by the AGREE II tool, was provided [14].

## Data Synthesis

A qualitative inductive thematic approach, drawing on Noblit and Hare’s stepped approach, was used to identify key concepts and synthesise the major themes [15], using the following three steps: (i) identifying original concepts and ideas from each paper that related to cross-cutting themes; (ii) synthesising these ideas into cross-cutting themes and (iii) identifying major themes. The review is registered on the international prospective register of systematic reviews (PROSPERO ID number CRD42024473285).

## Results

Of 809 articles identified through the database search, 94 were retained for full-text review. An additional 26 articles were added to the full-text review from the website search and other sources (i.e., citation search): a total of 120. After full-text review, 18 articles met the eligibility criteria **(**Fig. 1**).** The majority of the guidelines were developed in and for use in high-income countries [16–21], by multi-lateral organisations such as the WHO [22–24] or other international organisations often for their internal use [24–27] or for specific types of research (e.g. clinical trials, mental health research) [20, 25, 28]. Two guidelines focused on emergency settings with displaced populations and refugees [23, 25], with one of these focused specifically on the ethics of conducting mental health and psychosocial research (Table 1) [25]. Some of the migrant or refugee-specific guidelines considered in this review are working papers and do not include information on development, implementation and evaluation frameworks; thus, little is known on the operational framework and evaluation of these guidelines [16, 20, 29].

**Table 1:** Characteristics of the included guidelines

S. *N*	Title of the guideline	Year of publication /Latest Revision	Authorship/ Organisation	Country where the guideline was developed	Targeted population*	Settings**	Adult/ Paediatric^$^	Specific population or disease condition ^#^	Gender specific^β^
1	The National Statement on Ethical Conduct in Human Research [18]	2018	National Health and Medical Research Council (NHMRC)	Australia	General with mention of refugee population	Not specific	All ages with specific chapters for subgroups	Specific general recommendations for subgroups but not explicit reference to refugee/migrants	Specific section for pregnant and the human fetus
2	Code of ethics: Critical reflections on research ethics in situations of forced migration [33]	2018	International Association for the Study of Forced Migration	Canada	Forced migrant representing displaced population, refugees	Not specific	Adult	Not specific	Not specific
3	Guidelines for research with former refugees in New Zealand [16]	2008	Change makers-Refugees Forum	New Zealand	Refugee-like background	Resettled	Adult	Not specific	Not specific
4	Ethical guidelines for good research practice, United Kingdom [19]	2005	Adapted by the Refugee Studies Centre (RSC), Oxford University (United Kingdom)	United Kingdom	Refugee	Not specific	Adult	Not specific	Not specific
5	Report of the international bioethics committee on the bioethical response to the situation of refugees [17]	2017	International Bioethics Committee of the United Nations Educational, Scientific and Cultural Organisation (UNESCO)	Not specific	Migrant and refugees	Not specific	Adult	Not specific	Not specific
6	Guidance note: Research on refugees, asylum seekers & migrants [29]	2020	European Commission Directorate-General for Research and Innovation	European Union	Migrant, refugee and refugee-like background	Not specific	Adult	Not specific	Not specific
7	Guidelines funded through the Economic and Social Research Council Seminar Series ‘Eliciting the views of refugee people seeking asylum [32]	2006	Refugee and Asylum seeker Participatory Action Research (RAPAR), Economic and Social Research Council (ESRC), University of Salford, Revas	Not specific	Asylum seeker	Not specific	Adult	Not specific	Not specific
8	Research involving people of a refugee background: Considerations for ethical engagement [31]	2022	University college of Dublin	Ireland and Scotland	Refugee-like background	Not specific	Adult	Not specific	Not specific
9	Recommendation for conducting ethical mental health and psychological research in emergency settings [25]	2014	Inter-agency standing committee	Not specific	Displaced population	Emergency setting including the refugee setting	Adult	Mental health	Not specific
10	Ethical and safety recommendations for researching, documenting and monitoring sexual violence in emergencies [23]	2007	World Health Organization (WHO)	Not specific	Displaced population	Emergency setting including refugee settings	Adult	Specific section for people with cognitive impairment, an intellectual disability or a mental illness	Sexual violence
11	International Organisation of Migration (IOM)data protection manual [26]	2021	IOM	IOM specific	Migrants	Not specific	Adult	Not specific	Not specific
12	Participatory health research with migrants: A country implementation guide [22]	2022	WHO	WHO member states	Migrants	Not specific	Adult	Not specific	Not specific
13	International ethical guidelines for health-related research involving humans [30]	2016	Council for International Organisation of Medical Sciences in collaboration with WHO	Not specific	General with mention of refugee population	Not specific	Adult with specific guideline: for research among children and adolescents	Not specific	Specific section for women as participants
14	Advancing clinical trial engagement, involvement, and participation for people from culturally and linguistically diverse backgrounds [28]	2023	Australian Clinical Trials Alliance (ACTA)	Australia	Migrants (culturally and linguistically diverse population)	Not specific	Adult	Not specific	Not specific
15	Women’s Refugee Commission: Ethical Guidelines for Working with Displaced Populations through Programs, Research, and Media, 2023 [27]	2023	Women’s Refugee Commission	Not specific	Displaced women and children	Not specific	Adult with specific provision for children	Not specific	Not specific
16	Guidelines for Co-Produced Research with Refugees and Other People with Lived Experience of Displacement [21]	2023	University of New South Wales- Kaldor Centre for International Refugee Law, Act for Peace, Asia Pacific Network of Refugees, GAIN, R-SEAT, LERRN, Refugee-led Research Hub, Global Refugee-led Network, HERE, Amera International, University of Essex, RCAN, International Detention coalition, Mixed Migration Centre, Asia Pacific Refugee Rights Network, University of Auckland and Refugee Council of Australia	Australia	Refugee and displaced population	Not specific	Not specific	Not specific	Not specific
17	Framework for refugee and migrant health research in the WHO European Region [24]	2022	MacFarlane et al.	WHO European Region	Refugee and migrants	Not specific	Not specific	Not specific	Not specific
18	Community engagement and clinical trial diversity: Navigating barriers and co-designing solutions—A report from the “Health Equity through Diversity” seminar series [20]	2022	Reopell et al.	United States	Racial and ethnic minority populations	Not specific	Not specific	Not specific	Not specific

SN, serial number; * Target population seeks to identify Migrant/Refugee/ Refugee like background/internally displaced/mixed. ** Settings seeks to explore transit camps, detention centres and asylum set-up or resettlement in developed countries. $Targeted age groups: Children (0–5 years), adolescents (10–19), Adult (20–60), Ageing population ( 60+). # Specific condition such as mental health, paediatrics, people with disability. β Gender specific refers if the guideline was focused sexual assault or focusing on women or sexual and gender minorities- LGBTIQA+

All reviewed guidelines emphasise that general research ethics considerations are relevant to all human research undertakings [18, 30]. Additional considerations for migrants and refugees include:

### (i) Non-maleficence: Principle of “*do no harm*”

Care must be taken to anticipate, mitigate, and justify any harm caused to individuals, or communities, participating in research physically, psychologically, culturally, economically, or in any other way [16, 19, 22, 24, 26, 29, 31, 32]. Additional considerations posed by research with migrants and refugees include seeking assurance that participation will not influence housing, resettlement, relocation or visa status determination, nor lead to potential harm of participants’ family members, or communities (e.g. stigmatisation, loss of protection or potential identification) [17, 22, 23, 25, 29, 32]. Developing an additional layer of safety, close monitoring of participants and creating an enabling environment to address participants’ concerns were further highlighted [20].

As programmatic, research or media outputs may have detrimental effects on the immigration status, liberty, or safety of participants and their friends, families, and associates, data confidentiality and privacy are critical [26, 27]. Meaningful participation needs to be ensured across the research continuum from data collection, analysis and dissemination [31, 32]. Governance systems should be in place for authorisation for future use of data [30]. Researchers should anticipate problems that are likely to compromise anonymity such as small samples or specific groups, organisation or ethnic groups where it may be difficult to completely conceal the identity [19]. Privacy considerations need to be extended to include interpreters when used for participants with limited proficiency in the language used for recruitment and data collection, with awareness of the legislative and professional requirements for confidentiality, research integrity and risks of potential breaches [17, 27, 31, 33]. If an interpreter is required, it is important to allocate enough time for their involvement in the recruitment and data collection process [20]. Individual preferences need to be considered regarding the interpreter’s gender, background, and dialect and whether someone from the same cultural background is most appropriate [27]. In some situations, it may be more culturally appropriate to train local research partners to facilitate interviews or group discussions than researcher-led interpreted facilitation [27].

Several guidelines noted that particular care is required for individuals, families and communities of refugee background who may have a history of trauma, due to risks such as the potential for re-traumatisation [17, 25, 27, 31, 33]. In certain political contexts, some groups may be particularly vulnerable, especially small ethnic groups, socially stigmatised groups, religious minorities or people seeking asylum [19, 32]. In these circumstances, it may be advisable to aggregate data, withhold data from publication until the political or social context is less dangerous or refrain from cohort-specific research if normal research methods of de-identification and data protection cannot be embedded into protocols [19].

## (ii) Community Empowerment, Inclusivity and Reflexivity

Most guidelines emphasised the inclusion of relevant community partners throughout the research process, including design, data collection, analysis and dissemination of results [17, 20, 27, 31–33]. Reflexivity was considered as an important principle in research with refugees to dismantle power asymmetries and improve researchers’ accountability [21]. Enabling communities to actively participate in early decision-making around research priorities, policy, and practice through community-led engagement and involvement was also highlighted [22, 28]. Inclusion of relevant partners throughout the research process with appropriate protocols and mechanisms in place [20–22], co-creating a working agreement and recognising participants contractual interests and rights concerning data recordings and publication is recommended, acknowledging that rights will vary depending on the agreements and legal jurisdiction. Several guidelines included culturally appropriate conflict resolution mechanisms in case of disputes over methods, design, or dissemination [22, 27, 29, 31]. Six guidelines identified funding as critical to supporting participation in research with research applications being required to consider, and budget for: interpreters, transportation (where appropriate), extended clinic hours, community consultation and participation honorariums [16, 20, 21, 24, 27, 32]. Referral pathways and support systems during the research was also discussed by some guidelines [23, 25, 27].

## (iii) Social, Health and Scientific Value of Research

Most guidelines highlighted the need for research to be of relevance and significance to communities, and lead to the improvement of health knowledge nuanced for the population group when conducting research with refugees [16, 21, 23–25, 27, 29–31, 33] and/or migrants [22, 26]. Research findings should be shared with the participants themselves [22, 27, 31, 33] and dissemination facilitated through the inclusion of communities in developing dissemination plans [19, 32] whilst also conforming to data protection legislation [17, 26, 32]. Several guidelines recommend that researchers build on and collaborate with similar research to avoid over-researching or overburdening some populations [16, 31, 33].

### (iv) Respect for Rights and Dignity

Researchers should respect the autonomy of individuals in forced migration contexts and support their decision regarding their lives and participation in the research processes [16, 21, 24, 31–33]. The right of everyone including migrants and refugees to make their own decisions about their lives and degree of participation should not be overridden by the objectives of the research [32, 33]. The European Union “Guidance Note” recommends special protection to participants with diminished autonomy, such as unaccompanied minors, through engagement of non-governmental organisations or national authorities such as National Refugee Councils, with relevant experience, to provide legal advice, psychological support, language interpreting and/or legally appointed supervision [29]. Researchers should strive to protect the dignity of participants during data collection, analysis and publication [31]. This includes recognising that certain words or phrases may cause offence and should be avoided or, processes put in place to prevent undue distress to the participant and non-discriminatory language [32].

### (v) Cultural Awareness

Cultural awareness is stressed in most guidelines [16, 22, 27, 29, 31–33]. Language barriers may be mitigated through the use of an interpreter [17, 20, 22, 26–28, 31]. Contextual understandings, participant education levels, health literacy, gender, and broader cultural considerations may impact the understanding of the implications of participation in research and/or dissemination of findings [31, 33]. Cultural insiders may be helpful in mediating to ensure understanding of the research [29, 31], but are not always available or may not have the appropriate level of health or research literacy to support participants’ questions or engagement [31].

Most guidelines [16, 19, 22, 27, 29, 31, 33] discuss the importance of utilising culturally aware researchers well-versed in participants’ language and culture, and/or involving researchers familiar with the cultural beliefs and practices of the research participants was also discussed in few guidelines [17, 20]. Training researchers in cultural awareness is highlighted in four guidelines [22, 25, 27, 33]. Embedding learning and capacity building through a diverse workforce and work culture and establishing well-equipped and resourced diverse advisory groups, was highlighted in the clinical trials guideline by Australian Clinical Trials Alliance (ACTA) [28]. In the WHO country guideline for implementing participatory research with migrants, the authors cite a case study in which community interpreters successfully worked as peer researchers for migrant communities [17, 22]. International Bioethics Committee of the United Nations Educational, Scientific and Cultural Organisation (UNESCO) guidelines have recommended the use of professional interpreters as desirable, acting as a mediator and providing clarification concerning patient’s cultural values [17].

### (vi) Informed Consent

Informed consent is identified as a challenge by a number of guidelines [16, 19, 22, 23, 25, 27, 29, 32, 33]. Few guidelines [22, 27, 31], focused on the nature of the consent process with clarity, transparency and consideration of the participants’ circumstances. For especially-vulnerable populations, such as individuals in refugee camps or immigration processing facilities, or unaccompanied minors, it is advisable to ensure the presence of an experienced non-governmental organisation member with relevant and recognised credentials or a cultural insider during the informed consent process [27, 29]. Using oral consent processes (with approval from research ethics committee) may also help to overcome the challenge of signing consent [27, 29]. This also includes provisioning and funding for interpreter time, transportation, health literacy and trauma-informed approaches, which may necessitate several conversations (“iterative consent”) and/or slower paced discussion [17, 19, 20, 25, 27, 31, 32]. There are subgroups where consent is provided by parents or legal guardians, particularly for children, adolescents and those with cognitive incapacity [27]. When technical data-gathering devices such as audio/visual recorders and photographic records are being used, participants should be made aware of the capacities of such devices and records and be free to reject or modify their use [27].

### Quality Assessment of the Papers

The included guidelines had insufficient information to score in most of the domains of the AGREE II tool. Using the AGREE II assessment criteria across Domain 1 (*scope and purpose*), some guidelines did not clearly define the scope of the guidelines, including a lack of definition of the specific population for whom the guideline is intended [16, 29, 32, 33]. For Domain 2 (*stakeholder engagement)*, there was inadequate information on the views and preferences of the target population, type of stakeholders and target users [16, 17, 19, 29, 33]. Most guidelines did not have enough information in Domain 3 (*rigour of development*), including information on the systematic methods and procedures for updating the guidelines [17, 19, 25, 26, 29, 31–33]. Systematic methods for evidence synthesis were not reported in these guidelines [17, 19, 25, 26, 29, 31, 32]. Most documents though had a clearly outlined and easily identifiable set of recommendations (Domain 4), except for two of the guidelines [29, 32]. Only three of the guidelines provided some details on Domain 5 (*applicability of the guidelines*), mainly on the indicators and resources [22, 27, 28]. However, a detailed implementation plan, governance structure, facilitators and barriers for application and resources with an auditing process were not included in any of the guidelines. It was also difficult to ascertain the editorial independence for most of the guidelines.

## Discussion

Despite the enormous global numbers of migrants and refugees, there are limited dedicated national ethics guidelines available in English for research with people from migrant and refugee backgrounds across major Organisation for Economic Co-operation and Development (OECD) countries, including USA, UK, Australia, Canada and New Zealand, whichcomprise large proportions of migrants and resettled humanitarian refugees [16, 29, 33]. Terminology around asylum-seekers or migrants and their inclusion in guidelines varies internationally. The available guidelines focus strongly on refugees. While these populations instantiate many concerns around safety and consent, it would be helpful for the guidelines to also address specific categories such as documented and undocumented labour migrants, elders and young people working, travelling and studying.

The principles outlined in the reviewed guidelines are not necessarily focused on inclusive and collaborative research practices, but are primarily focused on not doing harm to participants [16, 27, 33]. Attention is paid to the “risks” faced by the population (e.g. intimate partner violence and sexual violence) [23, 27], and those being on the “outside” of the legal asylum or migrant system [32, 33]. Thus, research among migrants and refugees is often considered under the broad rubric of “research into marginalised and at risk” [18, 30]. Not only does this create a categoric assumption of vulnerability that may not apply to all migrants, it also may inadvertently lead to systemic exclusion of some groups of migrants and refugees from research [34], on the grounds of complexity and externally perceived risk to the participant [35].

Overall, there was a lack of nuanced discussion around community partnership, strategies to address intersectionality and decolonising research methodologies [36]. This review reveals the limited consideration given to the need for inclusive, collaborative and reciprocal research practices as a principle of good research [22, 29], which extends across the health continuum from quality assurance (e.g. retrospective audit) to clinical research. Gender minorities such as LGBTQIA+ communities may have faced multiple forms of violence during displacement. It is important to adopt an intersectional lens to research, policy and service to understand the different layers of discrimination that LGBTQIA+ refugees may face [37].

The need for translation of research outcomes to improve practice at all levels of clinical practice, health promotion and community engagement is also a recommendation that requires strengthening in guidelines. Furthermore, it is important to consider “waiver of the requirement of consent” (e.g. retrospective audit/quality improvement studies), and low risk audit research pathways that are equitable and include peoplewith migrant, refugee and refugee-like background rather than exclude such populations.

Informed consent is particularly important as a safeguard against the exploitation of participants that has occurred in the past [38]. Different ethnic groups have different concerns. Thus, variable approaches to recruitment and to the informed consent process are needed, which should be accepted and understood by ethics committees. For example, the need for a translated version of the consent was highlighted for individuals with limited proficiency in the language used for recruitment and/or the use of peer researchers and /or interpreters with relevant funding to support these needs [20, 27]. Considerations of accepting verbal consent are also important for participants who prefer verbal consent or those with limited literacy, to enable engagement and maintenance of the individual’s dignity [27, 29]. Awareness of vicarious trauma for some populations on mandating “*signatures for consent”* is important, given those that may be from asylum-seeker or refugee populations, who have faced previous persecution or torture. Vicarious trauma may also be present for interpreters in this context, as perceived compassion fatigue from continuous empathetic engagement with traumatic content [39], and should form part of guidelines, ensuring that there are mechanisms for debriefing and/or care escalation where required.

There is also sparse recognition of the unique needs of children and adolescents of migrant and refugee backgrounds. In 2019, Authors from Australia, outlined some of the legal and ethical issues regarding adolescent consent and confidentiality, consideration of adolescent development within a cultural context and refugee-related adversities, particularly for sensitive issues such as sexual/reproductive health, mental health issues, and undiagnosed neurodevelopmental disorders [40]. Competence to consent should follow the state’s laws, by the United Nations Convention on the Rights of the Child, and in many countries, as reflected in provisions around Gillick competence [41].

The other consideration with the research among the refugee, migrants or similar settings is the growing use of digital technologies to collect data. While, the use of technology can enable massive collation of data, this may also heighten the risk of breaching participants privacy, access-bias and even compromise informed consent if data are not properly collected or stored [42]. A nuanced approach to technology acceptance and ethical use of these technology in refugee or migrant research settings needs to be underlined [42, 43].

This review included guidelines available in English. The documents are the products of large non-government or multilateral transnational organisations, or high-income countries; we were unable to locate guidelines developed by institutions and organisations in lower- and middle-income countries, either because they were unavailable or not published in English. Our review benefited from broad and continuous stakeholder engagement through the establishment of the TAG comprising multidisciplinary expertise, people with lived experience and community-led organisations, which complemented the academic expertise oversight. This enhanced the refinement of search processes, awareness of grey literature, and informed the discussion and recommendations.

## Conclusion

There is national and international recognition of the need for guidelines to support the ethical conduct of research with people from migrant and refugee backgrounds. Such guidelines can prevent exacerbation of existing vulnerabilities and trauma, address community priorities and develop collaborative partnerships in research that build on community strengths. Research guidelines should address all considerations necessary for cultural safety, trauma-informed practice, inclusivity, prevent vicarious trauma, and minimise exclusion due to misconceptions about perceived risk. However, due safeguards must be in place, recognising both intersectionality and heterogeneity, as well as social determinants which influence consent, engagement, translation of outcomes and dissemination of findings.

## Supplementary Information

Below is the link to the electronic supplementary material.

Supplementary Material 1

## Data Availability

All data relevant to the study are included in the article or uploaded as supplementary material.
