# Supplementary material for: Ethical Conduct of Research with Migrants and Refugees: A Systematic Qualitative Review of Ethics Guidelines
Source: J Immigr Minor Health. 2026 Apr 14;28(3):702–15. doi: 10.1007/s10903-026-01894-z (PMC13222319; doi:10.1007/s10903-026-01894-z)
Supplement: Supplementary file 1 — Supplementary Material 1 [file 10903_2026_1894_MOESM1_ESM.docx]

**Ethical conduct of research with Migrants and Refugees:**

**A Systematic qualitative review of ethics guidelines**

**Supplementary Table S1: Search Strategy**

| **S.N** | **Search terms** | **# Hits** |
| --- | --- | --- |
| 1 | institutional ethics/ or ethics/ or research ethics/ or medical ethics/ | 66979 |
| 2 | ethic*.mp. [mp=title, book title, abstract, original title, name of substance word, subject heading word, floating sub-heading word, keyword heading word, organism supplementary concept word, protocol supplementary concept word, rare disease supplementary concept word, unique identifier, synonyms, population supplementary concept word, anatomy supplementary concept word] | 251941 |
| 3 | ((informed or presumed or shared or parent* or guardian* or family or families or child* or pediatric or paediatric or adolescent* or youth) adj2 (consent or choice or decision making or assent or dissent)).ti,ab,kf. | 73582 |
| 4 | (consent or assent or informed consent).tw. | 74048 |
| 5 | exp confidentiality/ | 58431 |
| 6 | guideline*.mp. [mp=title, book title, abstract, original title, name of substance word, subject heading word, floating sub-heading word, keyword heading word, organism supplementary concept word, protocol supplementary concept word, rare disease supplementary concept word, unique identifier, synonyms, population supplementary concept word, anatomy supplementary concept word] | 590920 |
| 7 | practice guideline/ | 33103 |
| 8 | consideration*.tw. | 327863 |
| 9 | (position statement* or policy statement* or practice parameter* or best practice*).ti,ab,kf. | 45529 |
| 10 | (Conduct adj2 research).tw. | 4857 |
| 11 | (Research adj2 complexities).tw. | 56 |
| 12 | recruitment.tw. | 153610 |
| 13 | (Resettlement or Unaccompanied minors).mp. | 2189 |
| 14 | exp refugee/ | 14858 |
| 15 | exp migrant/ | 15722 |
| 16 | Asylum seekers.mp. | 2158 |
| 17 | Refugee background.tw. | 155 |
| 18 | transient population.tw. | 256 |
| 19 | forced migrant.tw. | 29 |
| 20 | Humanitarian settings.tw. | 418 |
| 21 | immigrant.tw. | 15808 |
| 22 | (Culturally diverse population or (Culturally and linguistically diverse) or Limited English Proficiency or CALD).mp. or LEP.tw. [mp=title, book title, abstract, original title, name of substance word, subject heading word, floating sub-heading word, keyword heading word, organism supplementary concept word, protocol supplementary concept word, rare disease supplementary concept word, unique identifier, synonyms, population supplementary concept word, anatomy supplementary concept word] | 5441 |
| 23 | 1 or 2 or 3 or 4 or 5 | 369187 |
| 24 | 6 or 7 or 8 or 9 or 10 or 11 or 12 | 1078831 |
| 25 | 13 or 14 or 15 or 16 or 17 or 18 or 19 or 20 or 21 or 22 | 50290 |
| 26 | 23 and 24 and 25 | 262 |
| 27 | Limit to English | 248 |
